# A Novel CCM2 Gene Mutation Associated With Cerebral Cavernous Malformation

**DOI:** 10.3389/fneur.2020.00070

**Published:** 2020-02-07

**Authors:** Lipeng Yang, Jian Wu, Jing Zhang

**Affiliations:** Department of Neurology, Beijing Tsinghua Changgung Hospital, School of Clinical Medicine, Tsinghua University, Beijing, China

**Keywords:** cerebral cavernous malformation, gene mutation, CCM2, novel mutation, susceptibility-weighted imaging (SWI)

## Abstract

Cerebral cavernous malformations (CCMs) are the second most prevalent type of vascular malformation within the central nervous system. CCMs occur in two forms—sporadic and familial—the latter of which has an autosomal dominant mode of inheritance with incomplete penetrance and variable clinical expressivity. There are three genes considered to be associated with CCMs,—*CCM1*, which codes for KRIT1 protein; *CCM2*, which codes for MGC4607 protein; and *CCM3*, which codes for PDCD10 protein. To date, more than 74 gene mutations of CCM2 have been reported, and ~45% are deletion mutations. In this article, we disclose a novel *CCM2* genetic variant (c.755delC, p.S252fs^*^40X) identified in a Chinese family to enrich the database of CCM2 genotypes.

## Introduction

Cerebral cavernous malformations (CCMs) are the second most prevalent type of vascular malformation in the central nervous system (1). Histologically, the absence of pericytes and the deficiency of the tight and adherens junctions results in a single layer of endothelial cells, which leads to the impairment of the blood-brain barrier (BBB) (2). The incidence of CCM is estimated to be ~0.1–0.5% worldwide, but the data are underestimated because ~40% of patients are asymptomatic (3). CCMs can onset at any age but mostly occur between 20 and 50 years old (3, 4). The main clinical manifestations are intracerebral hemorrhage (ICH) and seizures, and other symptoms include recurrent headaches, focal neurological deficits, vertigo, and paresis (5, 6). MRI can be performed to diagnose CCMs. T2-weighted images can reveal the mulberry- or popcorn-like appearance and a dark rim due to hemosiderin deposition after repeated bleedings as the characteristics of CCMs. Furthermore, T2-weighted gradient echo sequences, which have more sensitivity to CMBs than T2-weighted images, can detect the small cerebral cavernomas (2, 7).

CCMs occur in two forms, a sporadic form and a familial form; the latter has an autosomal dominant mode of inheritance with incomplete penetrance and variable clinical expressivity. The proportion of familial cases was estimated as 50% in Hispanic-American patients (8). Compared to sporadic cases, familial cases are inclined to have multiple lesions (9). To date, there are three genes considered to be associated with CCMs, CCM1 (KRIT1, 7q11.2-q21), CCM2 (MGC4607, 7q15-p13) and CCM3 (PDCD10, 3q25.2-q27) (10, 11), which encode the proteins krev/rap1 interacting trapped 1 (Krit1), malcavernin, and programmed cell death 10 (PDCD10), respectively (10, 11). The three proteins constitute a heterotrimeric complex that plays an important role in endothelial cells and interacts with other signaling proteins (12).

Craig et al. (10) found that the mutation frequencies of the CCM1, CCM2, and CCM3 genes were 40, 20, and 40%, respectively, in 20 pedigrees of CCMs, and the penetrances were 88, 100, and 63%(13), respectively. Among these three genes, CCM2 used to be the only one with 100% of clinical and radiological penetrance (13). In 2018, however, Scimone et al. (14) found that penetrance of CCM2 was only 70% in a family with the novel mutation IVS10-1G>A.

In this study, we report a novel deletion mutation of the CCM2 gene in a Chinese patient with multiple CCMs, along with the clinical and neuroradiological features.

## Clinical Presentation

The patient is a 68-year-old Chinese woman who had a complaint of a 2-week vertigo with tinnitus and denied any headache, vomiting, diplopia, dysarthria, weakness or numbness in her limbs. She had intermittent vertigo for 4 years, hypertension for 4 years with untreated high blood pressure 160/100 mmHg, and hyperlipidemia for 2 years with statin treatment. The patient had lower extremity deep venous thrombosis 4 years ago with anticoagulation therapy for 1 month at that time. Before she came to our hospital, she had taken an aspirin anti-platelet therapy to prevent cerebral infarction for 2 months but stopped it half a month ago due to gastrointestinal discomfort. The physical examination found nothing abnormal but the right Babinski sign was suspiciously positive. However, when the patient underwent a head computed tomography (CT) scan and magnetic resonance imaging (MRI), cerebral hemorrhage and multiple cerebral microbleeds (CMBs) were found in her brain.

**Figure 1:**
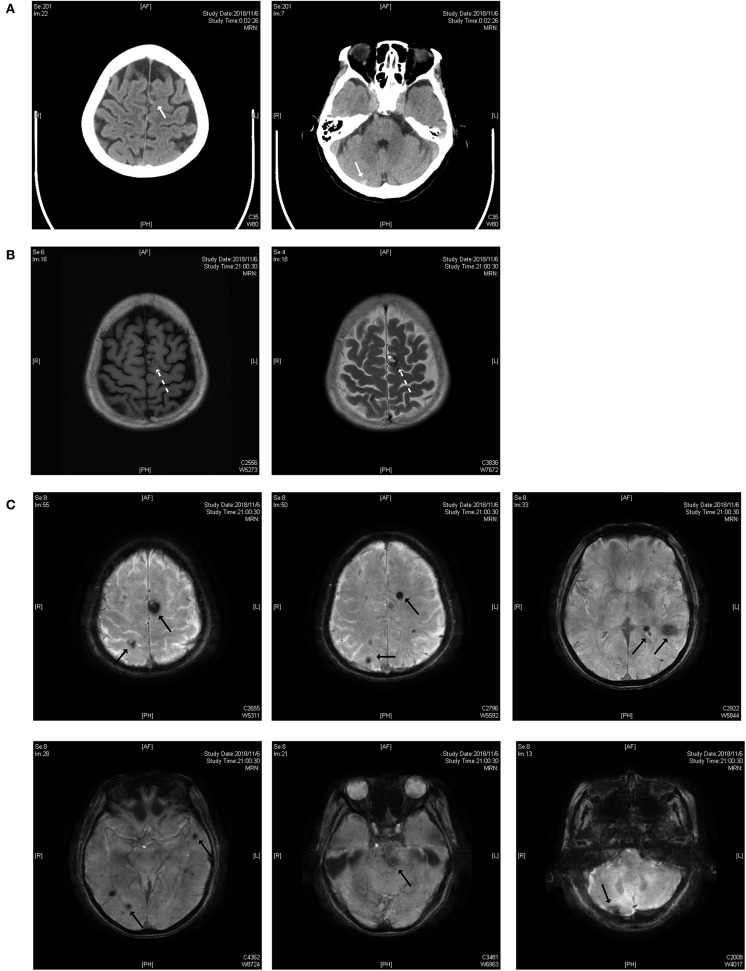
**(A)** Brain CT scan. The white solid arrows show a mixed-density lesion on the left parietal lobe and a high-density lesion on the right cerebellum. **(B)** T1 and T2 weighted images of the MRI scan. The dotted arrow indicates a popcorn-like mixed signal on T1WI and a high-signal lesion with a hemosiderin deposit surrounded on the T2 weighted image. **(C)** Susceptibility-weighted imaging (SWI). Black solid arrows indicate the lesions on the parietal lobe, temporal lobe, brainstem and cerebellum.

As shown in Figure 1, the lesions are located on the brainstem, cerebellum, temporal lobe, and parietal lobe. The patient had an acute hemorrhage on the lesion of the cerebellum, which may have caused her complaint of vertigo.

Digital subtraction angiography (DSA) found that the patient's right vertebral artery is posterior inferior cerebellar artery (PICA) type, without any vascular malformation. This examination ruled out other causes of hemorrhage, such as vascular malformation or aneurysm. Mini-Mental State Examination (MMSE) was normal with a score of 27. As to the treatment, we advised the patient to stay in bed and gave her Betahistine to relieve vertigo.

**Figure 2:**
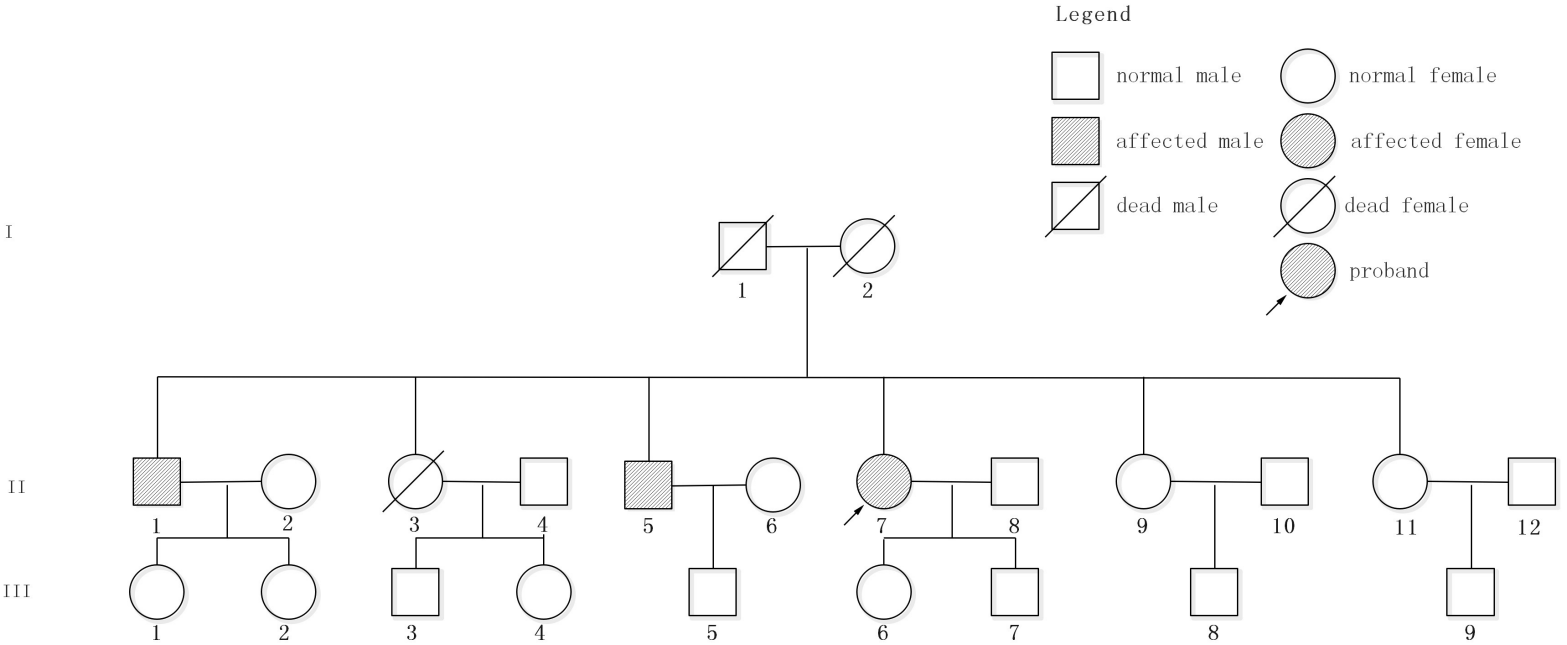
Pedigree of the studied family. Patient II7, indicated by the arrow, is the proband. II1 and II5 had cerebral hemorrhage without hypertension.

The patient had a family history of cerebral hemorrhage (Figure 2). She had two brothers and three sisters. The older brother (II 1) had a cerebral hemorrhage at 78 years old, with the symptoms of vertigo and nausea. There were multiple CMBs in the SWI of his head MRI. The younger brother (II 5) had a cerebral hemorrhage at 66 years old and his main symptom was also vertigo. Neither of them had hypertension. The proband has a daughter (48 years old now) and a son (43 years old now), and neither of them had cerebral hemorrhage. However, except for the proband, other family members denied the gene detection test.

## Laboratory Investigations and Diagnostic Tests

### Materials and Methods

Genomic DNA was extracted from whole blood of the subject using commercial kits and then stored at −20°C. Target acquisition technology was used to capture and enrich all the exons of the disease-related genes (CMM1, CCM2, and CCM3, included in a panel), including their upstream and downstream regions (~25 bp), and then they were sequenced on a high-throughput sequencer (Illumina). The DNA sequences were analyzed by Burrows-Wheeler Aligner (BWA) software. Single nucleotide polymorphisms (SNPs) and insertion-deletions (INDELs) were detected by Genome Analysis Tool Kit (GATK) software. The variants were confirmed by Sanger sequencing, and INDELs were analyzed by a deep algorithm based on the capture area. The variants were considered novel when they were not reported in previous publications or in the following public databases: (1) The Human Gene Mutation Database (HGMD) (http://www.hgmd.org/); (2) Exome Aggregation Consortium (ExAC) (http://exac.broadinstitute.org/); (3) National Center for Biotechnology Information (NCBI) dbSNP (http://www.ncbi.nlm.nih.gov/snp/); (4) ClinVar (https://www.ncbi.nlm.nih.gov/clinvar/); and (5) 1000 Genomes Project (http://www.internationalgenome.org/). The mutation was predicted by MutationTaster (http://www.mutationtaster.org/).

### Gene Analysis

**Figure 3:**
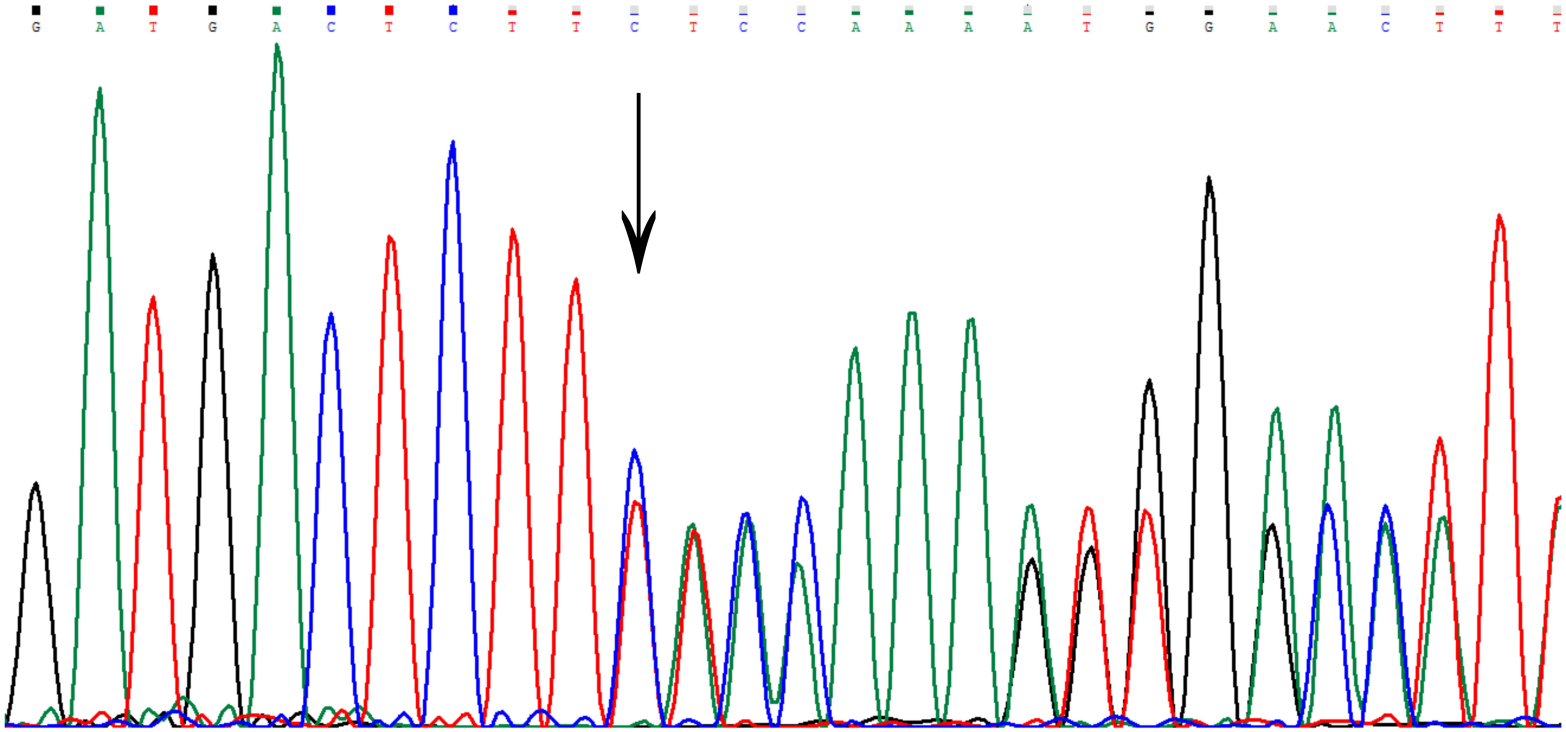
The genetic variant of c.755delC. The arrow indicates the deletion of a nucleotide at that site. There is a frameshift downstream of the deletion site.

Sequencing in patient II7 (Figure 2) revealed a novel variant in heterozygous condition, c.755delC (NM_031443.3 was selected as the reference sequence). The novel deletion (c.755delC) in the gene CCM2 exon 7 was a frameshift mutation that caused a premature stop codon at the 40^th^ amino acid downstream of the frameshift site (p.S252fs^*^40X), leading to a truncated MGC4607 protein that resulted in the loss of almost the entire C terminal domain (Figure 3). This variant has not been reported in previous publications or in the public databases mentioned above. CCM1 and CCM3 mutations were not found in the patient.

The mutation c.755delC is predicted to be disease-causing with a probability score of 1, which represents a high “security” of the prediction, by MutationTaster (http://www.mutationtaster.org/). Alignment of CCM2 protein sequences showed that the mutated site is on a very conserved sequence in different mammals, suggesting its importance to CCM2.

## Discussion

As discovered by previous studies, three CCM causative genes have been identified: CCM1/KRIT1, CCM2/MGC4607, and CCM3/PDCD10. The coding proteins constitute a CCM complex that has an important function in endothelial cells and is related to CCM pathogenesis.

**Figure 4:**
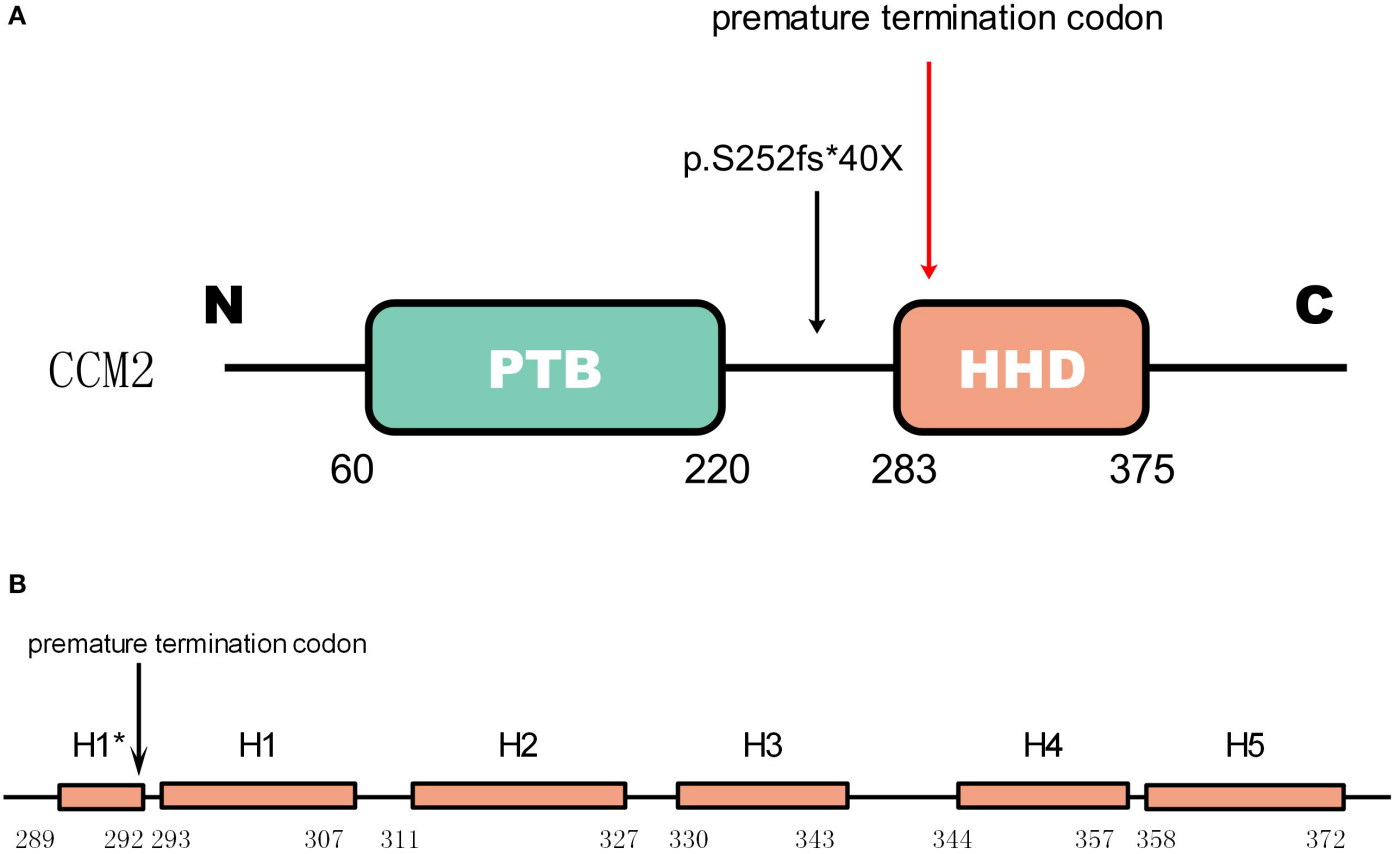
The mutation in CCM2 domains **(A)** shows the domains of CCM2. The green box represents the PTB domain [residues 60–220(15)]. The red box represents the HHD [residues 283–375(16)]. The mutation in the patient is indicated by a black arrow, and the premature termination codon (40 amino acids downstream) due to the frameshift is indicated by a red arrow. **(B)** Helices H1 to H5 with a 4-residue helix H1* (16). The premature termination site is indicated by the black arrow. This mutation causes a loss of almost all of the HHD.

Gene CCM2 located on chromosome 7 encodes protein malcavernin. This protein is a 444 amino acid protein predicted to contain a phosphotyrosine binding (PTB) domain at its N-terminus (15) and a harmonin-homology domain (HHD) at its C-terminus (15) (Figure 4). CCM2 interacts with CCM1 through the PTB domain via conserved NPxY/F motifs in the KRIT1 N-terminus (17) and directly interacts with the CCM3 C-terminus, where they stabilize each other (18). Therefore, CCM2 plays a bridge-like role in the CCM complex.

Recently Jiang et al. found that the CCM2 isoforms could be classified into two groups based on their alternative promoters and alternative start codon exons.They discovered a unique PTB domain, namely atypical phosphotyrosine binding (aPTB) domain. Both CCM1and CCM3 can bind competitively to this aPTB domain, suggesting CCM2 as a cornerstone for CCM signaling complex (19).

As reported by previous studies, a majority of mutations in these three genes result in truncations of their protein products, which leads to defective expression (20). As shown in Figure 4, for the newly discovered mutation p.S252fs^*^40X, the truncated protein product lost almost the whole HHD domain due to the premature termination codon.

In 2009, Harel et al. found that the C-terminus of CCM2 was linked to inducing cell death through a pathway involving the neurotrophin receptor TrkA (21). In 2013, Fisher et al. (16) found that the C-terminus of CCM2 can independently fold as a stable globular domain comprised of a five-helix bundle that they denote as helices H1 to H5 with a 4-residue helix (H1^*^). They found that there is distinct structural similarity to Usher syndrome scaffolding protein harmonin, a protein involved in mechanotransduction in hair cell bundles (22, 23). Therefore, they called this domain in CCM2 the harmonin-homology domain, or HHD (16). This domain interacts with MAP kinase kinase kinase (MEKK3) by binding to its N-terminus (24). Although disruption of this interaction does not influence MEKK3 catalytic activity, it indeed changes the subcellular localization of MEKK3 and then increases Rho/ROCK signaling (25). Furthermore, the disruption of their interaction increases the permeability of the neurovasculature *in vivo* (24), which may imply a possible mechanism of CCM with HHD damage.

The mutation p.S252fs^*^40X leads to the loss of all five helical bundles from H1 (residues 293-307) to H5 (residues 258-372), so we can predict that the abnormal product has a functional deficiency because of the loss of the HHD domain. Considering the function of HHD we mentioned before, it seems reasonable to predict that this mutation is a disease-causing mutation.

To date, more than 74 gene mutations of CCM2 have been reported, including nonsense, frameshift, splice mutations, and copy number variants (CNVs) (26). Approximately 45% are deletion mutations, which is consistent with the previous report published in 2007 but higher than the reported 38% (27). In conclusion, ~13 mutations (17% of total CCM2 mutations) were found in exons 7-10(28–33), which may impact the HHD. In contrast, almost 83% of mutations were located in exons 1-6, which may have an influence on the PTB domain. In Chinese population, there are about 10 CCM2 variants reported in familial and sporadic cases, 30% are delection, 20% are missense mutations, 20% are insertion mutations, and others are nonsense mutations, silent mutations and substitutions (1, 6, 34–36). Among them, 30% are located on HHD (1, 6, 35).

The relationship between phenotypes and genotypes should be studied further. Some CCM gene polymorphisms have significant associations with CCM symptomatology (37). Although the initial clinical symptoms are similar among the CCM1, CCM2, and CCM3 mutations, the CCM3 mutations appear to be related to more specific and severe phenotypes and earlier onset ages, while the CCM2 mutation carriers tend to have milder phenotypes (38). This finding may explain the mismatching of the relatively mild symptoms and the c.755delC mutation resulting in the nearly total loss of the HHD in this patient.

Family members of the patient refused to take gene examination, so we cannot provide more information about the penetrance of CCM2. As mentioned above, Scimone et al. found that the penetrance of CCM2 was 70% (14). He stated in the article that there were some carriers of IVS10-1G>A mutation, in age of 29–34,showing neither clinical symptoms nor cerebral cavernous malformations in MRI. One hypothesis was that they all in a young age compare to the disease's incidence age. But there were two family members with the similar situation, means they had same genotypes and similar age, whereas only one experienced the disease. Hence there may exist other genetic environmental factors that will affect the phenotype of CCM2.

## Conclusion

We report a case of CCM and its associated symptoms and genotypes. The deletion c.755delC is a novel mutation that has not been reported before or found in public databases. It is vital for researchers to determine the relationship between CCM phenotypes and genotypes and discover the role of CCM2 in the CCM complex.

## Data Availability Statement

Publicly available datasets were analyzed in this study. This data can be found here: http://www.hgmd.org/, http://exac.broadinstitute.org/, http://www.ncbi.nlm.nih.gov/snp/, https://www.ncbi.nlm.nih.gov/clinvar/, http://www.internationalgenome.org/.

## Ethics Statement

Written informed consent was obtained from the individual(s) for the publication of any potentially identifiable images or data included in this article.

## Author Contributions

LY designed the study, analyzed the data, and drafted the manuscript. JZ participated in the validation of the new mutation and revised the manuscript. JW participated in the analyses of the data and improved the study design.

### Conflict of Interest

The authors declare that the research was conducted in the absence of any commercial or financial relationships that could be construed as a potential conflict of interest.
